# Influence of Compost Amendments on Soil and Human Gastrointestinal Bacterial Communities during a Single Gardening Season

**DOI:** 10.3390/microorganisms12050928

**Published:** 2024-05-01

**Authors:** Sihan Bu, Alyssa W. Beavers, Kameron Y. Sugino, Sarah F. Keller, Katherine Alaimo, Sarah S. Comstock

**Affiliations:** 1 Department of Food Science and Human Nutrition, Michigan State University, East Lansing, MI 48824, USA; busihan@msu.edu (S.B.); kameron-sugino@ouhsc.edu (K.Y.S.); alaimo@msu.edu (K.A.); 2 Department of Nutrition and Food Science, Wayne State University, Detroit, MI 48201, USA

**Keywords:** soil, compost, gardening, microbiota, microbiome, human, bacteria, bacterial communities

## Abstract

To measure associations between gardening with different compost amendments and the human gut microbiota composition, gardeners (n = 25) were provided with one of three types of compost: chicken manure (CM), dairy manure and plant material (DMP), or plant-based (P). Stool samples were collected before gardening (T1), after compost amendment (T2), and at peak garden harvest (T3). Compost and soil samples were collected. DNA was extracted, 16S rRNA libraries were established, and libraries were sequenced by Illumina MiSeq. Sequences were processed using mothur, and data were analyzed in R software version 4.2.2. Fast expectation-maximization microbial source tracking analysis was used to determine stool bacteria sources. At T2/T3, the gut microbiotas of P participants had the lowest Shannon alpha diversity, which was also the trend at T1. In stool from T2, *Ruminococcus 1* were less abundant in the microbiotas of those using P compost as compared to those using CM or DMP. At T2, *Prevotella 9* had the highest abundance in the microbiotas of those using CM compost. In participants who used CM compost to amend their gardening plots, a larger proportion of the human stool bacteria were sourced from CM compared to soil. Soil exposure through gardening was associated with a small but detectable change in the gardeners’ gut microbiota composition. These results suggest that human interactions with soil through gardening could potentially impact health through alterations to the gut microbiota.

## 1. Introduction

The gut microbiota (the community of microorganisms, including bacteria, fungi, and viruses, that live within the digestive tract) are important for human health. While genetics influence the human gut microbiota to a small extent, environmental factors including diet and lifestyle are more influential [1]. The biodiversity hypothesis, an extension of the hygiene hypothesis, posits that human contact with environmental microbiota influences proper development of the immune system, and thus aids in the prevention of immune-mediated diseases [2]. This hypothesis has been supported by the increase in immune-mediated diseases that has occurred alongside the rise of urbanization and subsequent decrease in human interaction with environmental microbiota, the microorganisms that live in our surroundings [3,4].

Additional support for the role of environmental factors influencing the gut microbiota is found in studies that examine gut microbiome composition amongst groups that vary in geography and lifestyle. In a study examining the gut microbiota of urban and rural Russians, the gut microbiome of urban Russians was more similar to that of people living in Western countries than rural Russians, and gut microbial communities were more similar among individuals living within the same rural area [5]. Another study compared the gut microbiome of Nigerian urban-dwellers and rural subsistence farmers and found differences in the relative abundance of many bacterial taxa between the two groups, and several taxa that were unique to each group [6]. Research has also examined the relationship between quantity and/or type of greenspace and human microbiota alpha diversity. Alpha diversity quantifies the number of microbial taxa present and/or how even the abundances are among the taxa within a sample; higher microbiota diversity is frequently associated with better health [7,8]. A large multi-country study found that people with a high level of vegetation cover near their home had more diverse gut microbiota than those with a low amount of greenspace near their home [9].

One likely contributor to the differences seen in the aforementioned studies is the extent of exposure to, and the composition of, microorganisms found in soil. Soil has a diverse microbiota, and soil microbiota composition varies geographically [10,11]. There are several potential means for soil bacteria to impact the human microbiota, including inhaling soil dust through the nasal passages, and soil bacteria contacting the skin [12]. To test this experimentally, a pilot study was conducted in which intervention participants rubbed their hands with a soil mixture three times a day for two weeks. There was an increase in the alpha diversity of the intervention participants’ stool microbiome when compared with controls [13]. To further examine the potential for enhanced soil contact to impact the human microbiota, several studies have examined the effect of nature interventions on the microbiome of young children. For example, one study enhanced the biodiversity of a daycare yard by adding forest floor and sod. Compared with before the intervention, the relative abundance of *Clostridiales* and Shannon diversity of *Ruminococcaceae* in the children’s gut bacteria increased, and skin bacterial diversity increased [14]. In another study, a nature-exposure intervention for preschool children did not change the alpha diversity of the gut microbiota, but did change the prevalence of some taxa [15].

Gardening is a potential strategy for altering the human microbiota via direct contact with soil. It is a popular leisure activity for millions of Americans, as well as worldwide, and provides routine soil exposure even for urban dwellers. There are several pathways by which gardening could result in direct bacterial transfer from soil to humans, including when gardeners touch their mouths or food with dirt on their hands, inhaling dirt through the nasal passages, or consuming the foods harvested from the garden that might have soil or soil residue on them. To date, only a small number of studies have examined the association between gardening and human microbiota. One study found that immediately after gardening, there is a temporary increase in the number of soil bacteria taxa that are found on the skin of the hands, but that this change diminishes over a matter of hours [16]. Another study examined the gut microbiome across the gardening season, and found increases in some bacterial taxa at the peak of gardening season [17]. Beyond sole exposure to soil microbiota, gardeners may also experience exposure to microorganisms in compost, which typically consists of decomposed animal manure and/or plant material. The bacterial communities present in compost can vary widely. For example, one study found differing microbial community compositions among composted manure from different types of livestock [18]. Another study examined a variety of composts, with some solely consisting of plant-based material and others containing manure from various animals. This study found that while there are some bacterial taxa that are prevalent across different types of compost, there are distinct microbial communities based on compost type [19].

More studies examining how gardening influences the gut microbiome are needed, including on the potential for compost, a commonly used soil amendment, to influence the gut microbiome of gardeners. Our study examines the gut microbiome of gardeners at three time points across a five-month-long gardening season: prior to gardening, after adding compost to their garden plot, and at the peak of harvest. We aimed to (1) examine the gut microbiota composition and diversity across the three time points, and (2) to determine if soil or compost bacteria were present in the gardeners’ gut microbiomes.

## 2. Materials and Methods

### 2.1. Ethics Approval and Consent to Participate

This study was approved by the Michigan State University Institutional Review Board project #STUDY00002311: Your Garden and Your Gut initial approval: 8 April 2019. Prior to any study participation or data collection, participants completed written informed consent.

### 2.2. Study Design and Sample Collection

Potential participants were registered community gardeners gardening at one of the community gardens in the Greater Lansing Foodbank Garden Project network in metro Lansing, Michigan, or home gardeners registered to receive Garden Project support. Recruitment for this study occurred through attending garden orientation meetings and emailing enrolled gardeners. To be eligible for the study, participants were required to not have started gardening in 2019, not be pregnant, and be between the ages of 18 and 70 years old. Upon enrollment, participants completed a survey that included questions on demographics.

**Figure 1:**
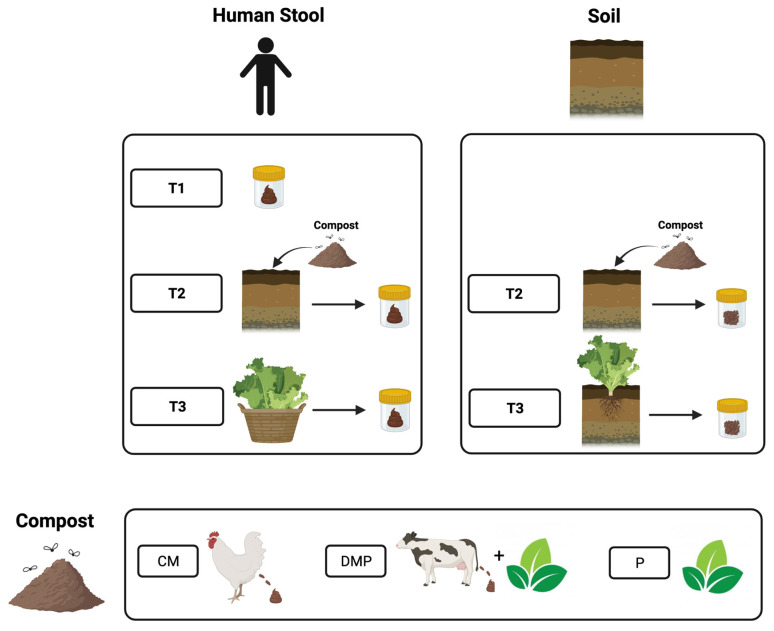
Study design. T1, time point 1; T2, time point 2; T3, time point 3; CM, chicken manure; DMP, dairy manure and plant-based materials; P, Plant material.

**Figure 2:**
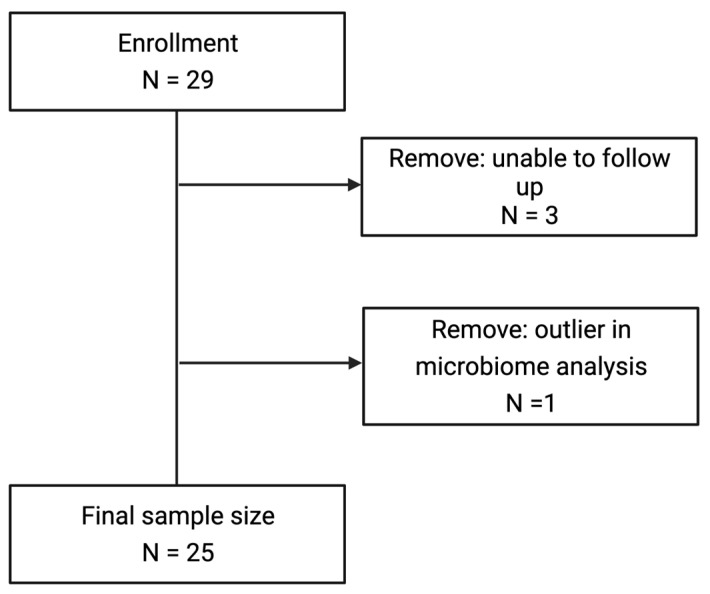
Flow chart of participants.

Participants were given one of three types of compost for their garden: pelletized chicken manure compost (CM), compost containing dairy manure and plant-based materials (DMP), or a compost comprised of only plant material (P) that contained food scraps and yard waste (Figure 1). Participants were instructed to spread the compost evenly over their garden plot and dig compost into the top 6 inches of soil. Participants were able to plant what they wanted in their garden plots. During the gardening season, participants collected their stool samples at three time points (Figure 1). Time point 1 (T1) was prior to beginning gardening in May or June. At time point 2 (T2), participants were instructed to work the provided compost into their garden plots and spend at least four cumulative hours gardening prior to collecting this stool sample. Time point 3 (T3) stool was collected during the peak of garden harvest, in late August or early September. Each time participants submitted a stool sample, they were asked if they were currently taking antibiotics. Soil samples from participants’ garden plots were collected by a researcher at two time points: time point 2 (T2) after participants amended their soil with compost, and time point 3 (T3) at the peak of the harvest in late August or early September. For each type of compost, three samples were collected by a researcher from each original batch of compost. Twenty-nine participants enrolled in the study, with three lost to follow-up and one outlier with a highly unique microbiome composition removed from the analysis, resulting in a final sample size of 25 (Figure 2).

### 2.3. DNA Extraction, 16S rRNA Gene Amplification, and Illumina Sequencing

Stool, soil, and compost samples were stored at −80 °C. DNA was extracted using the DNeasy PowerSoil Pro Kit (Qiagen MoBio, Carlsbad, CA, USA) following the manufacturer’s instructions. Due to the high level of diversity of bacteria in soil and compost, DNA was extracted from four replicates per sample. The four replicates were pooled together prior to PCR amplification. The V4 region of the 16S RNA gene was amplified using 515F/806R primers with barcodes SB501-SB508 and SA701-SA712 from IDT (Coralville, IA, USA) per the mothur wet lab SOP [20]. Thermocycler settings were as described previously [21]. The resulting amplicons (triplicates) were pooled and purified using Agencourt AMPure XP beads (A63880, Beckman Coulter, Brea, CA, USA) at a ratio of 0.7× beads volume to PCR product volume with two subsequent 70% ethanol washes prior to elution of 16S amplicons into molecular biology DNAse/RNAse-free water. The concentration of 16S rRNA gene amplicons was quantified using the Quant-IT dsDNA assay kit (Invitrogen, Carlsbad, CA, USA) after PCR purification. Purified 16S amplicons were pooled with the same amount of gDNA (ng) for each sample and sequenced at the Michigan State University Research Technology Support Facility with paired-end 250 base-pair sequencing using the Illumina MiSeq and standard V2 chemistry (Illumina, San Diego, CA, USA).

Sequence reads were processed in mothur following the Illumina MiSeq SOP [22]. After merging forward and reverse reads, sequences with ambiguous bases and any sequences longer than 275 bp were removed to ensure that sequences were of the V4 region. The SILVA reference taxonomy (release Version 132) was used to align the sequences. Sequences were classified by operational taxonomic unit (OTU) taxonomies using the SILVA reference. The reads were rarefied to the minimum number of reads, 14,549, prior to further analysis. For human stool, rarefaction curves were generated to confirm that sampling depth was adequate.

### 2.4. Statistical Analysis

The participants were classified into three groups according to the compost type they were assigned. One-way ANOVA (analysis of variance) was used to test whether the three groups differed in age and BMI (body mass index). Chi-square tests were used to examine if the three groups differed in race, sex, income, and baseline antibiotics use. Chao 1, inverse Simpson, and Shannon alpha diversity indices for all sample types (stool, soil, and compost) were calculated in R (version 4.2.2) using the vegan package [23,24]. Shapiro–Wilk tests were used to test if the alpha diversity data were normally distributed. Depending on the normality of the data, one-way ANOVA with Tukey HSD or Kruskal–Wallis with Dunn tests were used to examine differences in alpha diversity indices between the groups at a single time point. A Friedman test was used to measure the alpha diversity scores between the three time points.

For all of the sample types (stool, soil, and compost), Beta diversity (Sorensen and Bray-Curtis dissimilarity matrix) was calculated with the vegan package in R and ordinated using principal coordinate analysis (PCoA). Permutational multivariate analysis of variance (PERMANOVA) was performed to test the gut bacterial compositional differences between the groups at each time point based on the Bray–Curtis dissimilarity using the adonis function in the vegan package [25]. Individual taxa whose relative abundance was >1% were compared between groups using a negative binomial model in the MASS package [26] for independent (compost) groups analysis, and negative binomial mixed models or zero-inflated negative binomial mixed models in the NBZIMM package were used for dependent group comparisons (stool and soil) [27]. *p*-values from negative binomial models were FDR corrected with the Benjamini–Hochberg (BH) procedure. FEAST analysis was used to examine the possible sources of stool bacterial communities [28]. OTU count data was used as the input for the FEAST analysis. The type of compost the participant was assigned to was selected as the compost source in the FEAST analysis. For gardeners assigned to DMP or CM compost, a representative DMP or CM sample was used, since the DMP and CM composts were commercial products and were distributed to participants from the same batch. For gardeners assigned to P compost, the compost from their garden site was used as the source, since P compost was delivered in bulk to those gardens. *p*-values < 0.05 were significant.

## 3. Results

### 3.1. Participant Characteristics

Demographics overall and by compost type are presented in Table 1. We collected fecal samples from 25 participants with a mean age of 37.6 ± 10.3 years (mean ± SD). The average BMI was 27.3 ± 7.9 (mean ± SD). Most of the participants were white (n = 16) and female (n = 17) with a household income less than $50,000 (n = 14). There were two participants who reported currently taking antibiotics at T1, two participants at T2, and one participant at T3. Of the 25 participants, 9 were assigned CM compost, 10 were assigned DMP compost, and 6 were assigned P compost.

### 3.2. Alpha Diversity

The alpha diversity of the human stool samples was similar across the three time points (Table 2). Stool alpha diversity was also compared based on the compost type participants received, with statistical tests performed separately for each time point (Table 3). Participants using P compost had a significantly lower inverse Simpson score than those using CM at all three time points. Similarly, participants using P compost had a significantly lower Shannon score than those using CM at T2 and T3, and the Shannon score was nearly significantly different between these two compost types at T1 (*p*-value = 0.05). At each of the three time points, human stool Chao 1 indices were similar regardless of the compost group (Table 3). The alpha diversity of the compost bacterial communities and soil communities are shown in Supplementary Table S1. Alpha diversity significantly differed between the three types of compost for all three alpha diversity metrics. The alpha diversity of the soil bacterial communities at T2 and T3 were similar regardless of the compost type used to amend the soil (Supplementary Table S1).

### 3.3. Beta Diversity

**Figure 3:**
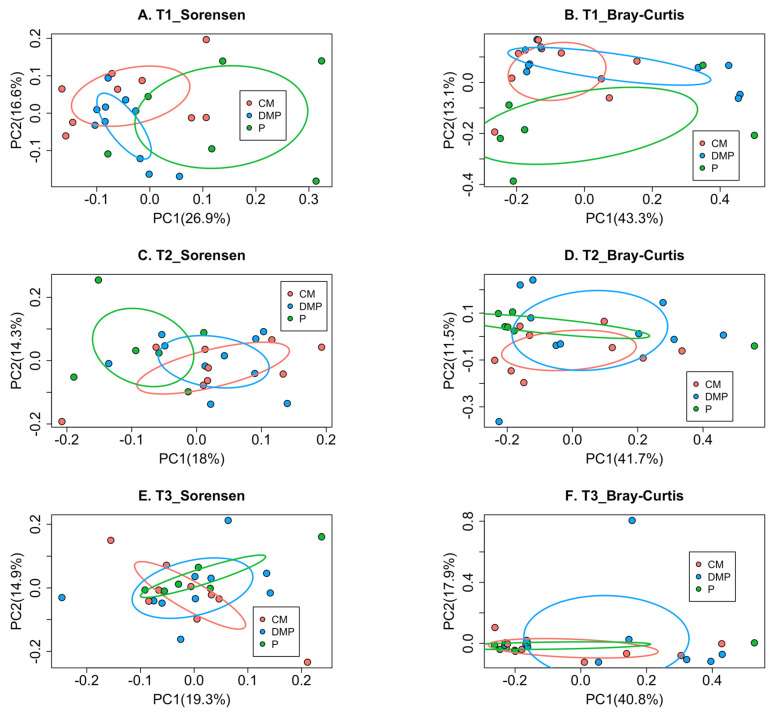
Beta diversity of human gut microbiota by compost types. Beta diversity at T1 (**A**,**B**), T2 (**C**,**D**), and T3 (**E**,**F**) is presented for membership (**A**,**C**,**E**) and community composition (**B**,**D**,**F**). *p*-values were (**A**) Sorensen, *p*-value < 0.01; (**B**) Bray-Curtis, *p*-value = 0.13; (**C**) Sorensen, *p*-value = 0.12; (**D**) Bray-Curtis, *p*-value = 0.53; (**E**) Sorensen, *p*-value = 0.53; (**F**) Bray-Curtis, *p*-value = 0.57. T1, time point 1; T2, time point 2; T3, time point 3; CM, chicken manure; DMP, dairy manure and plant-based materials; P, plant material.

**Figure 4:**
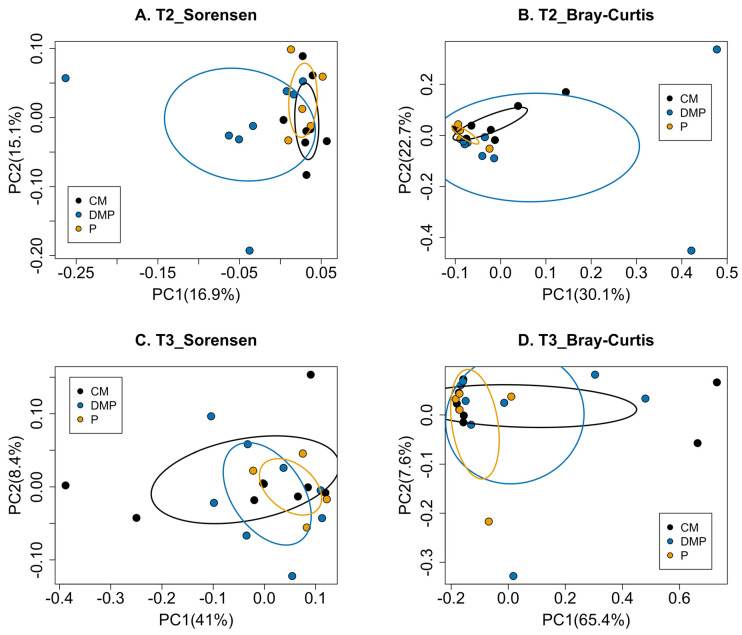
Beta diversity of soil bacteria by compost types. Beta diversity at T2 (**A**,**B**) and T3 (**C**,**D**) is presented for membership (**A**,**C**) and community composition (**B**,**D**). *p*-values were (**A**) Sorensen, *p* = 0.01; (**B**) Bray-Curtis, *p* = 0.06; (**C**) Sorensen, *p* = 0.42; (**D**) Bray-Curtis, *p* = 0.64. T1, time point 1; T2, time point 2; T3, time point 3; CM, chicken manure; DMP, dairy manure and plant-based materials; P, plant material.

At T1, prior to gardening, the human gut microbiota richness differed between the three compost groups (*p*-value < 0.01, Figure 3A); however, the bacterial community structure was similar (*p*-value = 0.13, Figure 3B). Neither the human gut bacterial membership nor composition differed at T2 (*p*-value = 0.12, Figure 3C; *p*-value = 0.53, Figure 3D) or T3 (*p*-value = 0.53, Figure 3E; *p*-value = 0.57, Figure 3F). At T2, the soil bacterial membership differed with respect to the compost type used to amend the soil in the gardening plot when beta diversity was assessed using the Sorensen index (*p*-value = 0.01, Figure 4A). However, the soil bacterial composition was similar when beta diversity was assessed using the Bray–Curtis dissimilarity matrix (*p*-value = 0.06, Figure 4B). There were no significant differences in membership by amendment type in soil samples collected at T3 for either the Sorenson (*p*-value = 0.42, Figure 4C) or Bray–Curtis (*p*-value = 0.64, Figure 4D) assessments.

### 3.4. Individual Taxa Differences

In the human stool samples, taxa with relative abundance greater than 1% were compared across the three time points. Only the significantly different taxa across time points are shown. When comparing the relative abundance of taxa across the three time points, we found that human stool samples at T2 had a significantly higher relative abundance of *Dorea* and *Ruminococcus 1* than those at T3 (Table 4). *Ruminococcus 1* was less abundant in T3 stool compared to T2 stool; however, *Collinsella* was more abundant in T1 stool. T1 stool had a higher abundance of *Dorea* and *Collinsella* compared to T3 stool.

We also compared taxa abundance between the three groups, stratified by time point (Table 5 for taxa with significant differences, Supplementary Table S2 for taxa with no significant differences). At both T1 and T2, *Prevotella 9* relative abundance significantly differed between all three groups, but the difference was no longer significant at T3. At T1, the relative abundances of *Coprococcus 2* and *Collinsella* were lower for participants who used the P compost compared to CM or DMP. *Ruminococcus 1* and *Ruminococcus 2* showed similar results: at T2, gardeners using P compost had the lowest abundance of these taxa compared to gardeners using DMP or CM compost. In addition, *Ruminococcus 2* was more abundant in T1 stool in the participants who used the DMP compost, compared to those who used CM or P. At T3, *Clostridium sensu stricto 1* existed at a higher relative abundance in the stool of those using P compost compared to those using CM or DMP compost (Table 5).

**Figure 5:**
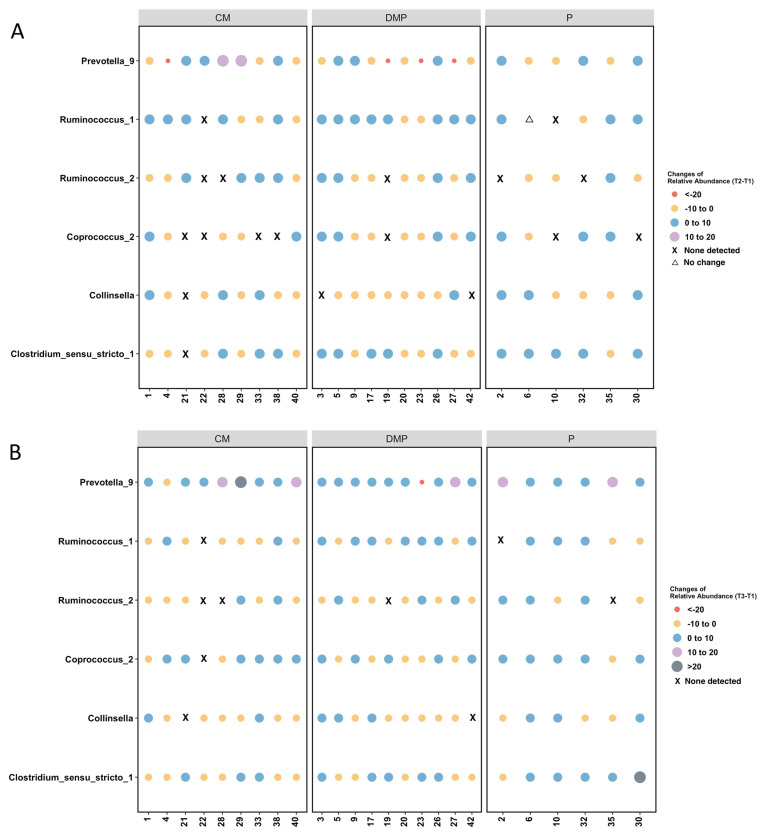

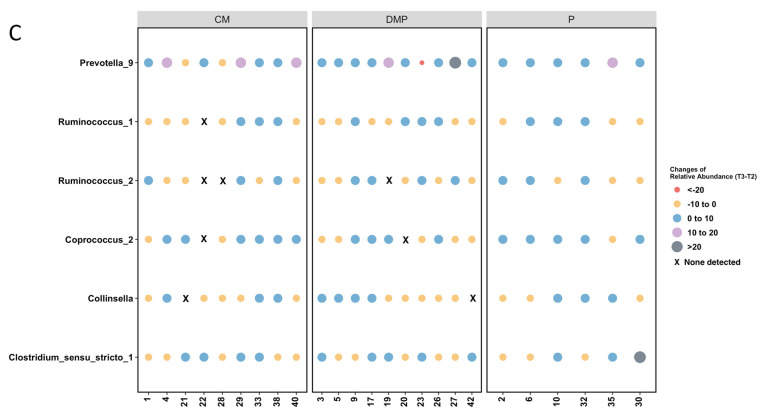
Individual participant taxa relative abundance changes. (**A**) Relative abundance changes from T1 stool to T2. (**B**) Relative abundance changes from T1 stool to T3. (**C**) Relative abundance changes from T2 stool to T3. Each column presents data for a single participant, with participants grouped by the type of compost they used. T1, time point 1; T2, time point 2; T3, time point 3.

Figure 5 presents the change in taxa relative abundance across time points, with data presented individually for each participant’s stool. The most consistent changes observed were in *Prevotella 9*. The relative abundance of *Prevotella 9* consistently increased from T1 to T3 and T2 to T3 in all of the participants who used P compost, and most participants who used CM or DMP compost.

For the soil samples, the taxa relative abundance at T2 and T3, stratified by type of compost used, was compared (Table 6). *SBR1031 ge, Veillonella*, and *Escherichia Shigella* had a higher abundance at T3 compared to T2 when the soil was amended using the P compost (Table 6). A numeric increase in *Veillonella* and *Escherichia Shigella* from T2 to T3 was observed in soil amended with CM. However, these increases were not observed in soil amended with DMP.

### 3.5. Source Tracking

**Figure 6:**
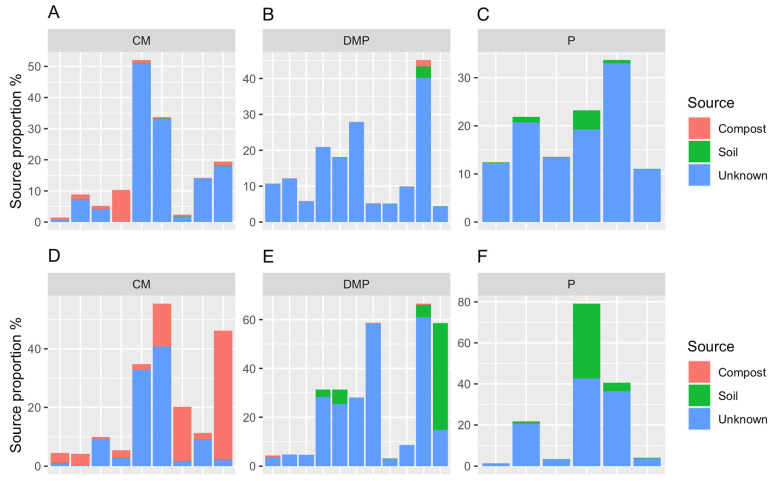
Bacterial 16S sequences in human stool collected at T2 and T3 that shared similarities with those from compost or soil. All non-accounted for source proportion originated from human stool 16S sequences at T1. Each bar is a separate participant and appears in the same order at T2 (**A**–**C**) and T3 (**D**–**F**). Thus, for each bar, 100% would be the participant’s total 16S sequences at either T2 (**A**–**C**) or T3 (**D**–**F**). T1, time point 1; T2, time point 2; T3, time point 3; CM, chicken manure; DMP, dairy manure and plant-based materials; P, plant material.

Using FEAST analysis and including the participant’s own T1 stool sample as a source, it was suggested that participants who used CM compost had a larger proportion of their gut bacteria potentially sourced from the compost compared with participants who used the other two types of compost (Figure 6). Conversely, participants who used DMP or P compost had a larger proportion of their stool bacteria that was potentially sourced from the soil compared with those using CM compost.

## 4. Discussion

This study investigated the association between soil and compost exposure and gardeners’ gut microbiota, as well as the potential for soil or compost bacteria to transmit to the human gut during gardening. The gut bacterial communities of participants who used P compost had lower Shannon alpha diversity compared to the gut bacterial communities of participants using the other two compost types after soil amendments (Table 3). In participants who used CM compost to amend their gardening plots, a larger proportion of the human stool bacteria were sourced from the compost compared to the soil (Figure 6). The results suggested that human interactions with soil through gardening could potentially impact health through alterations to the gut microbiota. However, direct evidence of improved health remains to be demonstrated.

The current study found that alpha diversity of the gardeners’ gut microbiota did not significantly change over the three time points (Table 2). This is similar to the findings from Brown et al. who showed that alpha diversity metrics of human fecal bacteria, including observed features and Faith’s phylogenetic diversity, were similar before the gardening season and during the harvesting season [17]. In our study, we found that participants who used P compost had a significantly lower richness (inverse Simpson index) at T1 and a significantly lower evenness and richness at T2 and T3 (Shannon and inverse Simpson indices) of bacteria than those who used CM compost (Table 3), suggesting that baseline differences in alpha diversity, and not the type of compost used, were responsible for the observed differences.

When examining specific taxa, we observed that some bacterial taxa significantly changed in gardeners’ stool over the gardening season (Table 4), though it must be acknowledged that factors like diet were not included in our models. Similarly, Brown et al. found that gardeners had significantly lower abundances of *Romboutsia* uncultured, *Terrisporobacter* uncultured, *Butyricicoccus* uncultured, and *Lachnospiraceae* cultured before the gardening season than at peak season [17]. In our study, an unclassified genus in the *Lachnospiraceae* family was significantly different over the three time points. However, the relative abundance of this taxa was similar between any of the two time points when tested using multiple pairwise comparisons. This might be due to the lack of statistical power when comparing people who used different types of compost. In our study, nine people used CM compost, ten used DMP compost, and six used P compost. However, three other taxa that significantly changed in the stools of gardeners over the gardening season (*Dorea, Ruminococcus 1,* and *Collinsella*) did have significant pairwise differences. *Dorea* is a gram-positive non-spore-forming bacteria that belongs to the *Lachnospiraceae* family and occurs in human feces [29]. In the current study, gardeners’ stools had a significantly lower relative abundance of *Dorea* at T3 than at T1 or T2. Compared to Italian urban-dwelling controls, a depletion of *Dorea* and unclassified *Lachnospiraceae* was found in Hadza hunter-gatherers’ fecal samples [30]. This suggests that these taxa may represent a key difference between industrialized-like and non-industrialized-like gut microbiomes [31]. Similarly, residents of Norman, Oklahoma who had an urban-industrialized lifestyle were enriched in *Dorea* compared to Matses and Tunapuco, individuals of a hunter-gatherer population and a traditional agricultural community, respectively [32]. This provides evidence that lifestyle is a key factor contributing to differences between industrialized-like and non-industrialized-like gut microbiomes. However, caution must be taken when drawing this conclusion since these analyses did not account for dietary intake by participants.

Compost houses a diverse microbiome, whose composition varies greatly by compost type [18,19]. In the present study, the three compost types had different alpha diversity of compost bacteria, but there was no association between soil alpha diversity and the type of compost with which the soil was amended (Supplementary Table S1). When examining the beta diversity of soil samples by type of compost, we found differences by compost type shortly after compost had been added to the soil (T2), but not later in the season (T3) (Figure 4). This result suggests that the soil bacteria composition was sensitive to compost additions [33,34,35], but that the compost’s effect on soil bacteria composition may have diminished over time [36]. Therefore, the environmental microbiomes gardeners are exposed to may differ by type of compost used, and the effects may vary across the gardening season.

According to the FEAST analysis, participants who applied CM compost to their gardens had more gut bacteria that were potentially transferred from the compost compared to those who used DMP or P compost (Figure 6). Conversely, participants who used DMP or P compost had more soil bacteria that was potentially transferred to their gut bacteria based on the results from the FEAST analysis. CM compost is comprised of only chicken manure, DMP is composed of both cow manure and plant material, and P is solely plant material. Chickens have a monogastric digestive system, which is also found in humans, while cows are ruminants. Thus, the chicken manure compost may contain more bacteria that is viable in the human gut compared to the DMP compost. Alternatively, chicken manure compost may harbor more similar bacteria to those found in the human gut. This requires further study.

In our study, there are some limitations that should be acknowledged. First, the sample size is relatively small, and may not have sufficient power to detect differences across the three time points. The sample size within each compost group is even smaller, resulting in low power to detect differences between compost groups. Thus, a larger sample size is necessary for future studies. Second, inclusion of a control group that does not garden would also be beneficial in order to examine if the gut microbiota changes over the seasons regardless of whether people garden. Each participant had a unique garden, so baseline soil differed by participant. Further, neither participant diet nor extent of time spent gardening or in the garden have been taken into account, and these factors should be analyzed in future research. In this study, only three time points across an entire gardening season were considered. Additional time points throughout the entire gardening season should be considered as the garden, the diet, and the gut are dynamic across time. Further, to truly determine bacterial transfer from soil/compost to the human gut, shotgun whole genome analysis of stool DNA is needed.

## 5. Conclusions

This pilot study is novel in that it investigated the association between gardening with different types of compost and the composition of the human gut microbiome. This study demonstrates the potential for soil and compost exposure to enable soil/compost-associated microbes to enter the human gut. Direct contact with soil for a duration of the gardening season might conclude with bacterial transmission from soil to gut, which then impacts gut microbial diversity and community composition with time. However, research describing soil and gut microbial interaction during gardening is lacking. Deepening our understanding of how environment microbial exposures shape our gut microbial diversity and community is needed. Such research can lead to more effective strategies for introducing new species to the gastrointestinal tracts of people at risk for disease or already impacted by disease.

## Figures and Tables

**Table 1:** Participant demographic characteristics.

Population Characteristics		Compost Type			
	All	CM	DMP	P	*p*-Value
N	25	9	10	6	
Continuous variables ^1^					
Age, years	37.6 ± 10.3	37.8 ± 12.6	34.6 ± 8.6	42.3 ± 8.8	0.36
BMI, kg/m ^2^	27.3 ± 7.9	28.7 ± 9.7	25 ± 5.8	29.2 ± 8.3	0.50
Categorical variables ^2^					
Race					0.25
Asian	7(28)	1(11)	5(50)	1(17)	
Black	2(8)	1(11)	0	1(17)	
White	16(64)	7(78)	5(50)	4(67)	
Sex					0.29
Female	17(68)	8(89)	6(60)	3(50)	
Male	8(32)	1(11)	4(40)	3(50)	
Income					0.59
Less than $50,000	14(56)	5(56)	7(70)	2(33)	
More than $50,000	10(40)	4(44)	3(30)	3(50)	
Antibiotics, Yes					——
T1	2(8)	0	0	2(33)	
T2	2(8)	0	1(10)	1(17)	
T3	1(4)	0	0	1(17)	

SD, standard deviation; T1, timepoint 1; T2, timepoint 2; T3, timepoint 3. ^1^ Continuous variables were calculated by one-way ANOVA. Data are presented as mean ± SD. ^2^ Categorical variables were calculated by Chi-square. Data are presented as N(%).

**Table 2:** Alpha diversity of human gut microbiota over the gardening season.

	T1	T2	T3	*p*-Value
Chao 1	121.9 ± 30.2	126 ± 28.1	144.3 ± 49.2	0.34
Shannon	2.8 ± 0.5	2.9 ± 0.4	2.8 ± 0.6	0.37
Inverse Simpson	10.7 ± 5.9	11.1 ± 4.7	10.2 ± 5.1	0.14

T1, time point 1; T2, time point 2; T3, time point 3. Friedman test was used to compare values of each alpha diversity index across timepoints. Values are Mean ± SD.

**Table 3:** Alpha diversity of human gut microbiota by compost type at each time point.

	T1				T2				T3			
Alpha Diversity	CM	DMP	P	*p*-Value	CM	DMP	P	*p*-Value	CM	DMP	P	*p*-Value
Chao1	128 ± 28.3	121.9 ± 26	112.6 ± 41.4	0.65	136.2 ± 38.3	122.8 ± 16.2	116 ± 25.3	0.37	164 ± 63	123.3 ± 20.9	149.7 ± 53.5	0.25
Shannon	3.2 ± 0.4	2.8 ± 0.5	2.5 ± 0.4	0.05	3.2 ± 0.3 ^a^	2.9 ± 0.3 ^ab^	2.6 ± 0.6 ^b^	0.02 *	3.1 ± 0.4 ^a^	2.8 ± 0.6 ^ab^	2.3 ± 0.7 ^b^	0.03 *
Inverse Simpson	14.5 ± 6.5 ^a^	9.4 ± 5.2 ^ab^	7.3 ± 3.1 ^b^	0.04 *	14.1 ± 4.8 ^a^	10.5 ± 4 ^ab^	7.8 ± 3.4 ^b^	0.02 *	12.7 ± 4.6 ^a^	10.4 ± 5.2 ^ab^	6.2 ± 3.2 ^b^	0.04 *

T1, time point 1; T2, time point 2; T3, time point 3; CM, chicken manure; DMP, dairy manure and plant-based materials; P, plant material. One-way ANOVA with Tukey HSD post-hoc test or Kruskal–Wallis with Dunn’s post-hoc test were used. Within each time point, values in a row that with different superscript letters (a, b) are significantly different, *p* < 0.05. Values are presented as mean ± SD. * *p*-value < 0.05 is significant.

**Table 4:** Human gut microbiota taxa that significantly changed over the gardening season.

	T1	T2	T3	*p*-Value
***Lachnospiraceae_unclassified***	2.30(1.6, 3.18)	3.11(2.13, 4.1)	2.41(1.34, 3.05)	0.04
***Dorea***	1.69(1.13, 2.08) ^a^	1.8(1.35, 2.27) ^a^	1.31(0.87, 1.65) ^b^	0.01
***Ruminococcus 1***	1.49(0.38, 2.52) ^ab^	1.75(0.01, 3.43) ^a^	0.98(0.05, 2.54) ^b^	0.03
***Collinsella***	0.78(0.43, 1.6) ^a^	0.56(0.08, 1.15) ^ab^	0.48(0.1, 0.87) ^b^	0.04

T1, time point 1; T2, time point 2; T3, time point 3. Negative binomial mixed models were used to examine relative abundance for bacterial taxa across three time points. Values are reported as medians (25th percentile, 75th percentile), and values in the same row with different superscript letters (a, b) are significantly different at *p* < 0.05. *p*-values were adjusted by BH procedure.

**Table 5:** Taxa differences in the human gut microbiota between compost types when stratifying by time point.

	T1				T2				T3			
	CM	DMP	P	*p*-Value	CM	DMP	P	*p*-Value	CM	DM	P	*p*-Value
***Prevotella 9***	1.69(0.02, 11.47) ^a^	0.04(0.01, 46.57) ^b^	0.02(0.02, 28.09) ^c^	<0.01 *	1.04(0.02, 17.7) ^a^	0.04(0.01, 30.17) ^b^	0.07(0.01, 27.56)^c^	<0.01 *	16.34(0.82, 20.43)	0.75(0.1, 33.72)	1.05(0.58, 39.73)	0.93
***Ruminococcus 1***	2.41(0.39, 2.68)	1.62(0.92, 3.18)	0.01(0.002, 0.46)	0.23	1.46(1.29, 2.81) ^a^	3.15(1.01, 5.14) ^b^	0.01(0.002, 1.72) ^c^	<0.01 *	1.22(0.22, 1.36)	2.63(0.77, 3.85)	0.4(0.02, 0.07)	0.26
***Ruminococcus 2***	1.09(0.55, 2.74) ^a^	0.94(0.58, 4.65) ^b^	0.003(0, 2.51) ^a^	<0.01 *	0.52(0.2, 3.47) ^a^	0.71(0.28, 5.01) ^b^	0.003(0, 2.38) ^c^	<0.01 *	0.52(0.02, 1.29)	1.3(0.31, 2.92)	0.06(0.02, 2.17)	0.26
***Coprococcus 2***	0.43(0, 2.43) ^b^	1.43(0.08, 3.33) ^c^	0(0, 0.005) ^a^	<0.01 *	0.12(0, 1.8)	1.16(0.09,1.93)	0.003(0, 0.01)	0.16	0.07(0.01, 0.84)	1.71(0.07, 2.69)	0.02(0.01, 0.03)	0.94
***Collinsella***	1.15(0.43, 1.6) ^ab^	0.98(0.30, 1.56) ^b^	0.70(0.49, 1.34) ^a^	0.01 *	——	——	——	——	——	——	——	——
***Clostridium*** ***sensu stricto 1***	——	——	——	——	——	——	——	——	0.17(0.01, 0.25) ^a^	0.04(0.01, 0.53) ^a^	0.27(0.16, 0.39) ^b^	0.01 *

T1, time point 1; T2, time point 2; T3, time point 3; CM, chicken manure; DMP, dairy manure and plant-based materials; P, plant material. Blanks (——) signify the taxa was not present at that time point, or the relative abundance was less than 1%. Negative binomial mixed models were used to examine the differences in relative abundance of the taxa between groups. Values are reported as medians (25th percentile, 75th percentile), and values in the same row with different superscript letters (a, b) are significantly different at *p* < 0.05. *p*-values were adjusted by BH procedure. * *p*-value < 0.05 is significant.

**Table 6:** Individual taxa differences in the soil bacteria by time points.

	CM			DMP			P		
	T2	T3	*p*-Value	T2	T3	*p*-Value	T2	T3	*p*-Value
***Subgroup_6_ge***	11.18(8, 13.64)	13.04(7.89,15.95)	0.96	12.09(7.13, 12.64)	9.46(8.22, 10.69)	0.98	11.95(10.27, 13.39)	12.48(12.39, 13.39)	0.55
***uncultured Gemmataceae***	1.76(1.35, 2.2)	2.18(1.55, 2.39)	0.96	2.04(1.65, 2.34)	1.68(1.23, 2.32)	0.98	2.15(1.74, 2.17)	2.4(2.21, 2.5)	0.26
***Bacteroides***	0.11(0.02, 0.46)	0.24(0.1, 1.09)	0.96	0.04(0.01, 0.19)	0.28(0.18, 0.91)	0.98	0.07(0.02, 0.3)	0.3(0.12, 0.64)	0.52
***WD2101_soil_group_ge***	1.28(0.93, 1.77)	1.7(0.69, 2.08)	0.96	1.19(0.96, 1.54)	0.88(0.75, 1.45)	0.98	1.3(1.28, 1.35)	1.37(1.23, 1.45)	0.67
***Bifidobacterium***	0.01(0, 0.1)	0.06(0.02, 6.45)	0.14	0.01(0.005, 0.07)	1.88(0.04, 8.53)	0.97	0.01(0.007, 0.03)	0.06(0.03, 0.58)	0.10
***SBR1031_ge***	0.4(0.34, 0.5)	0.6(0.37, 0.72)	0.63	1.63(0.69, 3.16)	1.74(0.73, 2.41)	0.97	0.36(0.31, 0.41)	0.71(0.59, 1.06)	0.03 *
***Hydrogenispora***	1.95(1.27, 2.48)	1.58(0.3, 2.35)	0.55	1.5(0.35, 2.41)	0.84(0.2, 1.42)	0.97	1.12(1, 2.14)	1.04(0.62, 1.32)	0.52
***KD4 96_ge***	1.19(0.76, 1.34)	1.24(0.79, 1.64)	0.96	1.23(0.94, 1.42)	0.96(0.89, 1.03)	0.97	1.39(1.15, 1.62)	1.39(1.03, 1.55)	0.64
***Lachnospiraceae_ge***	0.02(0, 0.2)	0.09(0.03, 2.75)	0.12	0.02(0.01, 0.04)	0.42(0.04, 3.68)	0.97	0.01(0.01, 0.04)	0.03(0.03, 0.54)	0.12
***uncultured_ge***	0.72(0.55, 1.39)	0.76(0.37, 1.06)	0.89	1.23(0.69, 1.59)	0.68(0.44, 1.16)	0.97	1.38(1.37, 1.54)	1.39(0.73, 1.72)	0.64
***Veillonella***	0.01(0, 0.03)	0.03(0.01, 3.01)	0.12	0.003(0, 0.05)	1.05(0.19, 3.26)	0.98	0.007(0.007, 0.1)	0.06(0.04, 0.25)	0.02 *
***Candidatus_Udaeobacter***	0.44(0.3, 0.5)	0.51(0.32, 0.86)	0.89	0.93(0.58, 1.7)	0.85(0.62, 0.9)	0.98	1.33(0.4, 1.53)	1.05(0.49, 1.63)	0.64
***Escherichia Shigella***	0.01(0.005, 0.09)	0.09(0.02, 3.22)	0.12	0.003(0, 0.02)	1.25(0.1, 3.77)	0.97	0.007(0, 0.01)	0.23(0.08, 0.54)	0.01 *

T1, time point 1; T2, time point 2; T3, time point 3; CM, chicken manure; DMP, dairy manure and plant-based materials; P, plant material. Negative binomial mixed models were used to examine the differences in relative abundance of the taxa between groups. Values are reported as medians (25th percentile, 75th percentile). *p*-values were adjusted by BH procedure. * *p*-value < 0.05 is significant.

## Data Availability

Data are available from the corresponding author. The data are not publicly available due to the small sample size and restrictions of participant consent forms.

## Supplementary Materials

The following supporting information can be downloaded at: https://www.mdpi.com/article/10.3390/microorganisms12050928/s1. Table S1: Alpha diversity of compost bacteria by compost type and soil bacteria by compost type. Table S2: Human gut microbiota taxa that were similar regardless of compost type at each timepoint.
