# Supplementary material for: Influence of Compost Amendments on Soil and Human Gastrointestinal Bacterial Communities during a Single Gardening Season
Source: Microorganisms. 2024 May 1;12(5):928. doi: 10.3390/microorganisms12050928 (PMC11123992; doi:10.3390/microorganisms12050928)
Supplement: Supplementary file 1 [file microorganisms-12-00928-s001.zip › microorganisms-2872593-supplementary.pdf]

Supplementary Table S1. Alpha diversity of compost bacteria by compost type and soil bacteria by compost type

	Compost Bacteria				Soil Bacteria (T2)				Soil Bacteria (T3)			
	Compost Type				Compost Type				Compost Type			
	**CM**	**DMP**	**P**	***P*-value**	**CM**	**DMP**	**P**	***P*-value**	**CM**	**DMP**	**P**	***P*-value**
**Chao1**	321.5±3.8[a]	775.4±41[b]	951.5±122.6[b]	<0.01	1006.7±227.4	984.5±146.5	1062.5±113.6	0.55	937.3 ± 278.8	950.8 ± 195.1	1016.9 ± 114.6	0.81
**Shannon**	3.6±0.4[a]	4.6±0.1[b]	4.9±0.3[b]	<0.01	4.9±0.9	4.8±0.6	5.2±0.2	0.49	4.5 ± 1.1	4.7 ± 0.6	5 ± 0.2	0.73
**Inverse Simpson**	18.2±7.4[a]	23.4±6.1[ab]	42.3±12.7[b]	0.04	42.8±27.9	37.6±19.9	46.8±14.2	0.63	32.2 ± 21.3	35.4 ± 21.9	38.9 ± 6.8	0.83

T2 Timepoint 2, T3 Timepoint 3, CM Chicken manure, DMP dairy manure and plant-based materials, P Plant material
Values are reported as Mean±SD
P < 0.05 is significant

Supplementary Table S2. Human gut microbiota taxa that were similar regardless of compost type at each timepoint.

	T1				T2				T3			
Taxa	CM	DMP	P	p-value	CM	DMP	P	p-value	CM	DMP	P	p-value
Lachnospiraceae_unclassified	3.9 ± 2.7	2.5 ± 1.8	2 ± 1	0.13	4.1 ± 2.4	3.4 ± 2.4	2.8 ± 1.7	0.83	3.5 ± 2.4	3.1 ± 2.9	1.4 ± 1.2	0.26
Bacteroides	13 ± 9.8	9.5 ± 7.2	9.5 ± 11.4	0.79	9.8 ± 5.6	10.1 ± 7.1	13.1 ± 14.1	0.94	12.1 ± 12.6	9.9 ± 6.9	13.6 ± 14.9	0.93
Faecalibacterium	7 ± 4.4	9.2 ± 3.3	3.1 ± 2.2	0.07	7.4 ± 3.9	8.7 ± 3.5	5.3 ± 2.8	0.76	7.4 ± 5.3	10.8 ± 4.1	5 ± 3.7	0.42
Bifidobacterium	1.3 ± 1.4	3.4 ± 5	2.2 ± 4	0.53	1.8 ± 2	2 ± 2.5	1.8 ± 2.6	0.99	2.3 ± 1.5	1.8 ± 1.7	5.3 ± 8.1	0.42
Blautia	14.5 ± 5.9	9.6 ± 5.2	14.7 ± 10.2	0.26	14.3 ± 6.1	10.7 ± 5.2	12 ± 8.8	0.83	10.9 ± 4.4	11.2 ± 5.8	8 ± 7	0.93
Lachnospiraceae_ge	3.3 ± 2	3.8 ± 2.2	6.4 ± 3.4	0.13	3.6 ± 1.3	3.9 ± 2.5	4.8 ± 1.9	0.83	2.7 ± 0.9	3.7 ± 2	3.3 ± 3.1	0.93
Subdoligranulum	2.5 ± 1.3	2.2 ± 1.9	2.2 ± 3	0.96	3 ± 2	2.7 ± 2.1	1.7 ± 2.5	0.83	2.3 ± 1.4	2.3 ± 1.5	1.7 ± 2.5	0.93
Agathobacter	3.4 ± 2.8	2.4 ± 2.1	4.9 ± 4.3	0.41	4.8 ± 5	3.7 ± 2.7	3.4 ± 2.4	0.93	3.9 ± 2.8	3.1 ± 1.9	2.6 ± 2.6	0.93
Fusicatenibacter	2.6 ± 1.4	2.9 ± 2.2	4.1 ± 5.2	0.73	3.3 ± 2.7	3.2 ± 2.2	4.3 ± 5.1	0.94	2.4 ± 1.8	3.3 ± 2.8	1.8 ± 2.2	0.93
Anaerostipes	1.7 ± 1.1	0.9 ± 0.7	3 ± 2.4	0.05	2.2 ± 1.7	1 ± 0.7	2.9 ± 2.5	0.16	1.5 ± 1.1	1.3 ± 0.8	1.2 ± 1.2	0.93
Dorea	2.3 ± 1.3	1.4 ± 0.5	1.7 ± 1.4	0.41	2.4 ± 1	1.7 ± 0.7	1.3 ± 1.1	0.62	1.6 ± 0.5	1.1 ± 0.4	0.9 ± 0.5	0.26
Phascolarctobacterium	2.5 ± 2.2	3.1 ± 2.3	1 ± 1.4	0.41	2.1 ± 1.5	2.2 ± 2	1.2 ± 1.6	0.93	2.5 ± 2.3	2.4 ± 2.1	3.1 ± 4.3	0.94

T1 Timepoint 1, T2 Timepoint 2, T3 Timepoint 3, CM Chicken manure, DMP dairy manure and plant-based materials, P Plant material.
Values are reported as Mean±SD
P-values were adjusted by BH procedure
